# Polygenic Index for Sleep Duration and Brain Changes over Time

**DOI:** 10.3390/medsci14010088

**Published:** 2026-02-13

**Authors:** Tsapanou Angeliki, Chapman Silvia, Lee Seonjoo, Habeck Christian, Gu Yian, Stern Yaakov

**Affiliations:** Cognitive Neuroscience Division, Department of Neurology, Columbia University, New York, NY 10032, USA; sc4056@cumc.columbia.edu (C.S.); sl3670@cumc.columbia.edu (L.S.); ch629@cumc.columbia.edu (H.C.); yg2121@cumc.columbia.edu (G.Y.); ys11@columbia.edu (S.Y.)

**Keywords:** sleep, polygenic index, brain morphometry

## Abstract

Background: Sleep is a complex physiological process, crucial for cognitive functioning, emotional regulation, and overall health. Recent advances in genomics and neuroimaging have illuminated the intricate relationship between genetics, sleep architecture, and brain changes. This study investigated the association between sleep duration genetics, through a Sleep Duration Polygenic Index (Sleep PGI), and brain changes (total cortical thickness, white matter volume, gray matter volume, white matter hyperintensities volume) in cognitively healthy adults aged 20–80 years old. Methods: Using longitudinal data from the Reference Ability Neural Network (RANN) and Cognitive Reserve (CR) studies, we examined the impact of Sleep PGI on brain measures (total cortical thickness, gray matter volume, white matter volume, WMH volume) over time. Generalized Estimated Equations were used for the statistical analysis. Analysis was performed in the total sample (*n* = 94) and in three age-groups (young, middle, old). Results: Across age, higher Sleep PGI was associated with higher temporal WMH volumes over time. In models considering an interaction of age between Sleep PGI and time in study, age emerged as a significant moderator for outcomes of hippocampal volume, cortical white matter volume, and WMH volume (total, parietal) as outcomes. Conclusions: Sleep duration polygenic score was associated with changes in the brain in cognitively healthy adults. Genetic predisposition for longer sleep duration was associated with more favorable longitudinal trajectories against brain decline, a result mostly driven by younger adults. These findings underscore the importance of maintaining optimal sleep duration and the potential for personalized interventions to improve sleep and brain health.

## 1. Introduction

Sleep is a complex physiological process that is critical for cognitive functioning, emotional regulation, and overall health [1]. Recent advances in genomics and neuroimaging have begun to elucidate the relationship between genetics, sleep architecture, and brain changes [2].

Emerging research has consistently highlighted a significant association between sleep duration and brain changes [3], underscoring the vital role that sleep plays in neural health and cognitive function. Studies employing neuroimaging techniques, such as magnetic resonance imaging (MRI), have shown that both short and excessively long sleep duration is linked to adverse brain outcomes, including reductions in grey matter volume in areas critical for cognitive processing, emotional regulation, and memory consolidation [4,5,6,7]. Specifically, insufficient sleep has been correlated with atrophy in the frontal cortex and hippocampus, regions that are implicated in executive function and memory, respectively [8,9]. Conversely, prolonged sleep duration has been associated with similar neuroanatomical changes, suggesting that an optimal sleep window is crucial for maintaining brain structure integrity [3,10]. These findings are pivotal, as they suggest that sleep duration can directly influence brain health, potentially via mechanisms involving synaptic pruning, neurogenesis, and the clearance of neurotoxic waste, which are critical for neural plasticity and overall brain function [11,12]. Thus, maintaining optimal sleep duration emerges as a key factor in preserving cognitive abilities and preventing neurodegenerative changes.

Genome-wide association studies (GWAS) have identified multiple loci associated with various sleep characteristics, including sleep duration, efficiency, latency, and architecture, highlighting the heritable nature of sleep [13,14]. Specific genes, such as those involved in the regulation of circadian rhythms (e.g., CLOCK, PER2) and sleep homeostasis (e.g., DEC2, ABCC9), have been linked to distinct sleep phenotypes [15], suggesting that genetic predispositions can influence sleep quality and quantity [16,17]. The association between sleep genetics and brain changes is a dynamic field that offers insights into the biological underpinnings of sleep and its critical role in mental health and brain aging. While most previous research has relied on self-reported or actigraphy-based measures of sleep duration, recent genomic approaches provide an opportunity to explore the heritable basis of sleep traits. In this context, the Sleep Duration Polygenic Index (Sleep PGI) serves as a genetic proxy for individual differences in habitual sleep duration. The Sleep PGI is derived from GWAS that aggregates the effects of multiple common genetic variants associated with self-reported sleep duration. Although it does not capture actual sleep behavior or duration per se, it reflects inherited predispositions toward shorter or longer habitual sleep, offering a stable and lifelong marker without measurement noise or daily fluctuations. As such, while indirect, the Sleep PGI provides a useful tool for investigating genetically influenced sleep traits in relation to long-term brain health.

Further, neuroimaging studies have revealed that variations in sleep architecture, influenced by genetic factors, are associated with differences in brain morphology and activity [18]. For example, sleep spindles, which are regulated by genetic factors, have been correlated with increased grey matter density in brain regions involved in learning and memory, such as the hippocampus and frontal cortex [19,20,21]. Additionally, genetic predispositions to shorter sleep duration have been linked to alterations in brain structures associated with cognitive and emotional processes, indicating a potential genetic basis for the association between sleep and neuropsychiatric disorders [22].

The association between sleep genetics and brain changes is a dynamic field that offers insights into the biological underpinnings of sleep and its critical role in mental health and brain aging. In the current study we investigated the association between sleep duration genetics, through a Sleep PGI, and brain changes over time, in cognitively healthy adults across the adult age-range. We hypothesized that a higher genetic propensity for longer sleep duration—as indexed by the Sleep PGI—will be associated with more favorable longitudinal brain trajectories, reflected in slower rates of age-related brain structural changes over time, especially driven by younger adults.

## 2. Methods

Participants: Study participants were recruited for the Reference Ability Neural Network (RANN) and the Cognitive Reserve (CR) study. The RANN study was designed to identify networks of brain activity uniquely associated with performance across adulthood for each of the four following cognitive abilities: memory, fluid reasoning, speed of processing, and language [23]. The CR study was designed to elucidate the neural underpinnings of cognitive reserve and the concept of brain reserve [24]. All participants were native English speakers, right-handed, with at least a fourth-grade reading level. In order to be included in the study, participants had to be also free of any major neurological or psychiatric conditions that could affect their cognition. Careful screening excluded participants with Mild Cognitive Impairment (MCI) or Alzheimer’s disease Dementia (AD). Additional inclusion criteria for participants required (1) a score equal or greater than 130 on the Mattis Dementia Rating Scale [25], in order to guarantee a cognitively normal status; (2) minimal or no functional capacity complaints [26]; and (3) complete data on imputed genome-wide genotyped (GWAS), sleep, cognitive performance in all domains, and socio-demographic variables (sex, age, and education). Both RANN and CR studies have been approved by the Institutional Review Board of Columbia University. More detailed information about the two studies can be found in previous publications [23,27,28].

Genome-wide single nucleotide polymorphism genotype data (GWAS): Each participant had venous blood drawn during their visit at Columbia University. DNA samples were obtained through whole blood extraction. Genotyping was performed using Omni 1M chips, according to Illumina procedures. Genotype calling was performed using GenomeStudio v.1.0. Quality control was applied to both DNA samples and SNPs. Specifically, samples were removed from further analysis if they had call rates below 95%, sex discrepancies, and relatedness.

GWAS imputation: GWAS data for all study participants was imputed using the Haplotype Reference Consortium (HRC v1.1) panel through the Michigan Imputation online server [29]. The HRC is a reference panel of 64,976 human haplotypes at 39,235,157 SNPs constructed using whole genome sequence data from 20 studies of predominantly European ancestry [30].

Polygenic Index (PGI): Using the summary data of the GWAS of sleep duration reported by Dashti et al. [13], we derived a Polygenic Index (PGI) based on 78 SNPs in our sample. SNPs reaching genome-wide significance in the discovery GWAS and passing standard quality control procedures were selected. Of these, 78 SNPs were available and well-imputed in the present dataset and were therefore included in the final PGI. PRSice software v2 [31] was used to construct the PGI and to graphically display the PS-phenotype association results. Further details can be found in previous publication of our group [32].

Principal Components: Principal Components (PCs) of genetic variance were calculated using plink version 1.9 and were added in the models as covariates to control for potential population substructure [33], cryptic relatedness [34], and batch effects [35]. PCs might be related to the outcome but are independent of the PGI.

Imaging data: All scans were acquired on the same 3.0 Tesla Philips Achieva MRI scanner. Out of the brain measures used based on previous publications and on the theoretical importance on aging, we incorporated the following measurements: total cortical thickness, gray matter volume, white matter volume, and white matter hyperintensities. A T1-weighted Magnetization Prepared Rapid Acquisition Gradient Echo (MPRAGE) scan was acquired with an Echo Time/Repetition Time (TE/TR) of 3/6.5 ms and flip angle of 8°, in-plane resolution of 256 × 256, field of view of 25.6 × 25.6 cm^2^, and 165–180 slices in the axial direction with a slice thickness/gap of 1/0 mm. FreeSurfer (v5.1.0) software for human brain imaging analysis (http://surfer.nmr.mgh.harvard.edu/ accessed on 15 June 2024) was used for the reconstruction of the T1 scans [36,37]. WMH: FLAIR images for visualization of WMH were used with the following parameters: Repetition Time/Inversion Time (TR/TI) (ms) 11,000/2800 TE (ms): 125, in-plane resolution 256–189, FOV 23.0–17.96 cm, and 30 slices with slice thickness/gap of 4/0.5 mm. For the extraction of the WMH we used the Lesion Segmentation Tool, which is a toolbox for Statistical Parametric Mapping, able to segment T2 hyperintense lesions in FLAIR images. Lesions were segmented by the lesion growth algorithm [38] as implemented in the Lesion Segmentation Tool version 2.0.15 (https://www.applied-statistics.de/lst.html accessed on 15 June 2024) for Statistical Parametric Mapping.

### Statistical Analysis

Statistical analysis was performed using SPSS v29 (SPSS, Chicago, IL, USA) [39]. To examine the association between Sleep PGI and brain changes over time, we used generalized estimating equations (GEE) with Gaussian distribution. GEE takes into account correlated datapoints due to repeated measurements over time. The repeated measures for each subject are treated as a cluster [40]. A total of eight GEE models included the brain measures as the dependent variable, with time (years), Sleep PGI, and an interaction term between time and Sleep PGI as predictors. Models were adjusted for age group (young, middle, and old), sex, education, and the first four PCs of the SNP data and intracranial volume (ICV) as independent variables. A significant interaction term indicates a differential brain change over time as a function of the Sleep PGI. In a second set of models, we further included the three-way interactions of time, PGI, and age group (young: 20–44 y.o., middle: 45–64 y.o., old: 65–80 y.o.) to examine the moderating role of age on PGI sleep and changes in brain integrity over a 5-year follow-up. When the time × PGI × age group interactions were significant, we performed post hoc contrast analysis to quantify the effect of PGI on the change in outcomes by age group. To ensure robustness of the findings, we performed sensitivity analyses using GEE with log-normal distribution for the WMH measures.

## 3. Results

In total, 94 participants were included in the study. The parent study had a total of 471 individuals enrolled with available brain measures at baseline. Out of these, a total of 169 individuals had Sleep PGI and PCs scores processed, only 96 identified as white and had a follow-up visit, and 2 individuals were missing neuroimaging data, which determined our final sample size. Baseline demographic characteristics as well as sleep genetics and time in study are reported in Table 1 for total sample and age groups. There were no significant differences observed in main demographics, time in study, or Sleep PGI score other than the expected age difference groups (see Table 1). Brain imaging data available by interval is reported in Supplementary Table S1 by total sample and age group.

**Figure 1:**
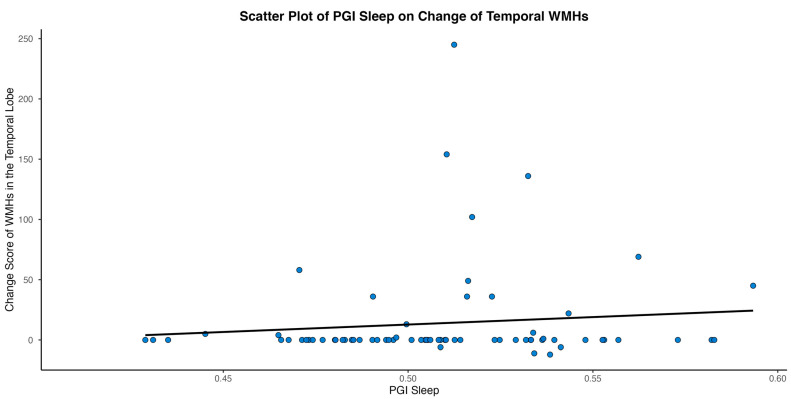
Individual-level change score in temporal white matter hyperintensity (WMH) volume over time plotted against Sleep Duration Polygenic Index (Sleep PGI). For visualization purposes, change score reflects unadjusted change in WMH from interval 1 to 2. Change therefore reflects WMH at time 2 subtracted from WMH at time 1. Positive values therefore reflect increases in WMHs.

Initial analysis including the total sample covering the whole adult age range showed that higher Sleep PGI was associated with higher Temporal WMH volumes over time (see Figure 1, Table 2). This result remained significant after transforming temporal white matter hyperintensity volumes into a log normal distribution. None of the other associations between Sleep PGI and the rest of the brain measures were significant across age.

**Figure 2:**
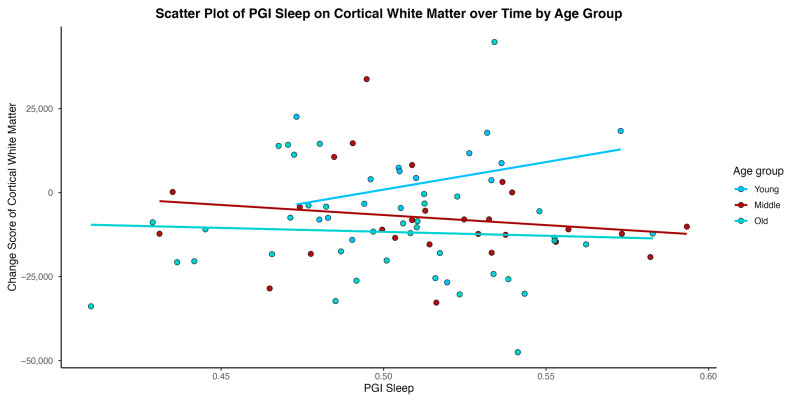
Individual-level change score in cortical white matter volume over time plotted against Sleep Duration Polygenic Index (Sleep PGI). Change Scores are reflective of unadjusted change between intervals 2 and 1 as described in the statistical analyses section.

**Figure 3:**
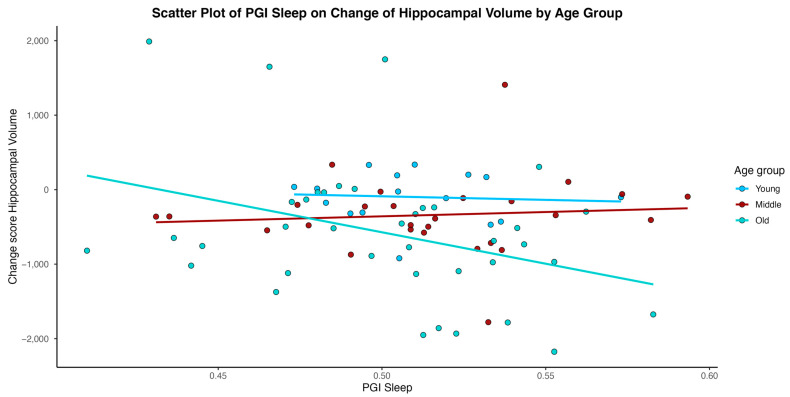
Individual-level change score in hippocampal volume over time plotted against Sleep Duration Polygenic Index (Sleep PGI). Change Scores are reflective of unadjusted change between intervals 2 and 1 as described in the statistical analyses section.

**Figure 4:**
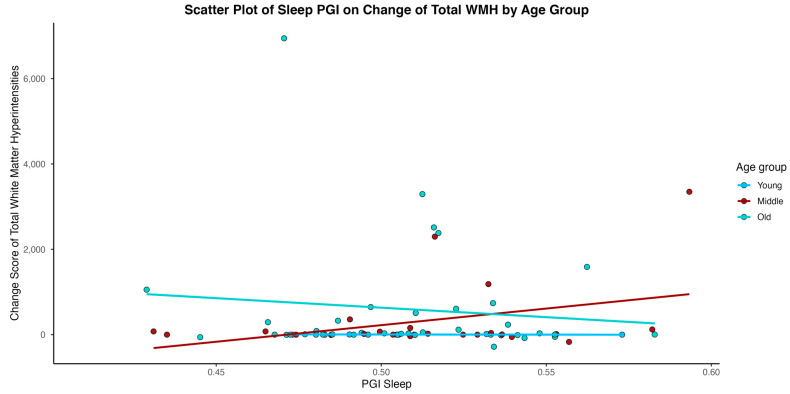
Individual-level change score in total white matter hyperintensity (WMH) volume over time plotted against Sleep Duration Polygenic Index (Sleep PGI).

**Figure 5:**
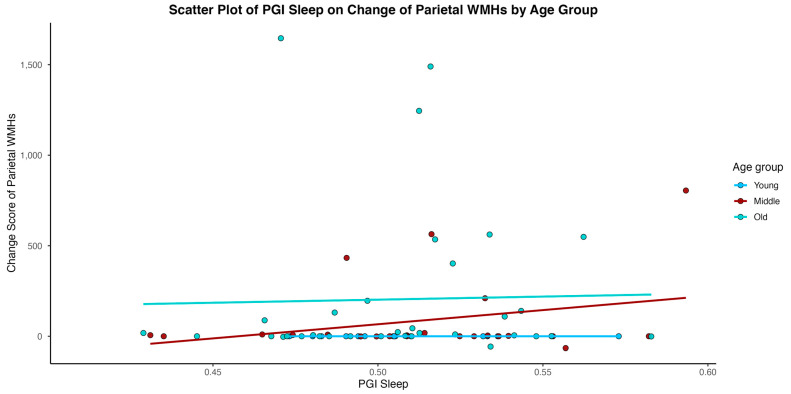
Individual-level change score in parietal White matter hyperintensities volume over time plotted against Sleep Duration Polygenic Index (Sleep PGI).

In models considering an interaction of age between Sleep PGI and time in study, age emerged as a significant moderator for the outcomes of hippocampal volume, cortical white matter volume, and WMH volume (total, parietal) (See Table 3). As depicted in Figure 2, Figure 3, Figure 4 and Figure 5, younger individuals experienced a lower differentia rate of change in brain metrics of cortical white matter, hippocampal volume, and reduced parietal WMH relative to older adults. In cortical white matter differences were also observed between young and middle-aged adults, with younger adults exhibiting a lower differential rate of change (see also Table 4 for contrast estimates of each age group). While the scatterplots depict raw unadjusted differences for visualization, the reported statistical significance is derived strictly from GEE models that mathematically account for the variable time intervals. Supplementary analyses for WMH metrics transformed for a log normal distribution showed that only the results of WMH in the parietal lobe remained significant. Please see Supplementary Table S2.

## 4. Discussion

The initial analysis of the full sample (ages 20–80) revealed a significant association between Sleep PGI and longitudinal changes in white matter hyperintensities volume in the temporal lobe. Specifically, individuals with higher Sleep PGI exhibited lower differential rate of change in regional WMH volume over time. This finding aligns with the existing literature linking sleep disturbances—such as obstructive sleep apnea—to WMH, suggesting that improving sleep quality may help preserve white matter integrity [41]. Importantly, this association reflects average differences in longitudinal slopes, rather than uniform change across individuals. White matter plays a crucial role in facilitating communication between brain regions [42]. Interestingly, our results build upon previous findings that objectively measured long sleep duration is associated with lower fractional anisotropy and higher radial diffusivity in white matter tracts [10,43]. However, most prior studies have focused on narrow age ranges, limiting their generalizability. In contrast, our findings extend across the adult lifespan.

In the age-specific analysis, results revealed a significant association between the Sleep PGI and specific brain volumes, including the hippocampus and white matter, as well as regional white matter hyperintensities, particularly total and parietal WMH volume. These findings may indicate that younger individuals with a genetic predisposition for longer sleep duration tend to show more favorable brain outcomes in certain regions. The stronger associations observed in younger adults may reflect a period of relative neurobiological stability during which genetic influences on brain structure are more readily detectable, rather than protection against aging-related decline [44].

In early to mid-adulthood, brain structure is largely maintained and less affected by heterogeneous vascular or neurodegenerative processes, allowing genetically driven inter-individual differences to be more visible [45,46]. With increasing age, accumulating pathology and environmental influences may reduce the relative contribution of genetic factors to observed brain changes [47,48]. Genetic influences on sleep-related traits may be more pronounced earlier in adulthood and attenuate with advancing age as environmental exposures and aging-related processes increasingly shape brain structure. As individuals age, the brain undergoes a range of structural and functional changes [46]. Environmental exposures, lifestyle factors, and age-related neurodegenerative processes become increasingly influential [49]. Large-scale developmental neuroimaging work indicates that indices such as cortical thickness, surface area, and related structural features show nonlinear trajectories across adolescence into early adulthood, and that “apparent stability” over short follow-up intervals can mask meaningful developmental heterogeneity that depends on where an individual falls along these trajectories [50]. These accumulating external and internal influences may attenuate or overshadow the impact of sleep-related genetic factors on brain morphology in older adults. These findings do not imply the existence of a critical or sensitive period but rather highlight age as a potential moderator of genetic associations with brain integrity [51].

Rather than implying structural protection, the observed associations may reflect brain characteristics that support functional maintenance through adaptive or compensatory mechanisms. Contemporary models of brain reserve emphasize that preserved cognitive function often arises from dynamic network reorganization, including interhemispheric interactions, rather than from static preservation of brain structure alone [52]. Recent work highlighting compensatory interhemispheric connectivity in aging and injury contexts supports this framework and provides a biologically plausible context for the present findings [46,47,53,54,55].

There are some limitations to consider. The main limitation of the study is the relatively small sample size, representative of only non-Hispanic white race and ethnicity, which may reduce the generalizability of the results and limit the statistical power [56]. The sample size may limit sensitivity to detect small genetic effects and complex interactions, and that replication in larger, independent longitudinal cohorts will be essential to confirm the robustness and generalizability of the present findings. Analyses were limited to individuals of European genetic ancestry, consistent with the ancestry of the GWAS discovery samples used to construct the Sleep PGI. This constraint reflects limitations in polygenic index transferability across ancestries rather than race or ethnicity per se and may limit generalizability to other populations. Polygenic indices often show reduced predictive accuracy across ancestries, underscoring the need for future studies in more diverse cohorts to validate the generalizability of sleep–brain genetic associations. A further limitation is that visual inspection of individual-level change scores may not fully reflect the population-level associations estimated by longitudinal models. Given the cognitively healthy nature of the sample and the relatively short follow-up interval, absolute changes in brain measures were small for many participants [57]. Further, younger and middle-aged adults, who are more likely to be employed or responsible for caregiving, may experience chronic societal sleep restriction that limits their ability to express genetically influenced sleep tendencies. As actual sleep duration was not directly assessed in the present study, we cannot determine the extent to which sleep behavior aligned with genetic predisposition across age groups [58]. Lastly, sleep disorders were not formally assessed in this cohort, and therefore the potential influence of undiagnosed or subclinical sleep conditions cannot be excluded [59]. Despite these limitations, the study has several notable strengths. Most importantly, it employs a longitudinal design, allowing for the observation of changes over time [60]. It also includes comprehensive MRI data, covering multiple brain volumes. Finally, the sample spans a wide adult age range, rather than being limited to a specific age group.

The findings underscore the importance of considering age when examining the impact of genetic factors on brain health. There may be an age-related pattern in how sleep-related genetic predispositions associate with brain structure, particularly during middle age, though further research is needed.

## Figures and Tables

**Table 1:** Baseline characteristics of our sample, in total and by age group.

	Total	Young	Middle	Old	Omnibus Test *p*-Value
Age, years, Mean (SD)	58.2 (15.3)	30.7 (5.9)	58.3 (5.6)	70.1 (3.8)	<0.001
Sex, women, *n* (%)	45 (47.9%)	8 (44.4%)	16 (44.4%)	19 (45.2%)	0.921
Education, years, Mean (SD)	16.5 (2.3)	16.4 (2.5)	16.2 (2.0)	16.7 (2.4)	0.687
Time in study (years)	4.4	4.5	4.3	4.4	0.469
PGI sleep	0.5	0.5	0.5	0.5	0.195
Total, *n*	94	18	36	42	-

**Table 2:** Parameter coefficients of the interaction between Time and Sleep PGI in the GEE models adjusted for age, sex, education, ICV, 4 PCs.

Brain Measure	Parameters			
		*β*	95% CI	*p*
Total Cortical Thickness	Time × PGI whole group	−0.005	−0.056, 0.046	0.847
Cortical Grey matter volume	Time × PGI whole group	3025.972	−22,694.334, 28,748.2478	0.818
Subcortical grey matter volume	Time × PGI whole group	−6587.740	−17,186.406, 4010.927	0.223
Cortical White matter volume	Time × PGI whole group	2204.305	−23,625.548, 28,034.158	0.867
Hippocampal * volume	Time × PGI whole group	−646.262	−1675.046, 382.522	0.218
Total White Matter Hyperintensities	Time × PGI whole group	791.435	−1296.572, 2879.443	0.458
Temporal White * Matter Hyperintensities	Time × PGI whole group	**102.903**	**19.219**, **186.588**	**0.016**
Parietal White Matter Hyperintensities	Time × PGI whole group	407.006	−105.848, 919.860	0.120

* Significant age group *Sleep PGI* years in study interaction. Bolded: Significant parameters.

**Table 3:** Parameter coefficients of interaction time*Sleep PGI*age group in the GEE models adjusted for age, sex, education, ICV, 4 PCs, time × age, time × sleep time × 4 PCs.

Brain Measure	Parameters			
		*β*	95% CI	*p*
**Total Cortical Thickness**	Time × PGI × Age group old	0.003	−0.007, 0.013	0.523
	Time × PGI × Age group middle	0.004	−0.009, 0.017	0.588
Cortical Grey matter volume	Time × PGI × Age group old	−2002.530	−8004.423, 3999.363	0.513
	Time × PGI × Age group middle	3.499	−6886.253, 6893.250	0.999
Subcortical grey matter volume	Time × PGI × Age group old	−1621.207	−3732.397, 489.983	0.132
	Time × PGI × Age group middle	−652.740	−2804.819, 1499.338	0.552
White matter volume *	**Time × PGI × Age group old**	−**6697.461**	−**10,766.856**, −**2628.066**	**0.001**
	**Time × PGI × Age group middle**	−**5281.885**	−**10,127.561**, −**436.208**	**0.033**
Hippocampal * volume	**Time × PGI × Age group old**	−**234.183**	−**395.567**, −**72.799**	**0.004**
	Time × PGI × Age group middle	−84.353	−245.640, 76.934	0.305
Total White Matter Hyperintensities * ^	**Time × PGI × Age group old**	**166.149**	**28.897**, **303.400**	**0.018**
	Time × PGI × Age group middle	111.977	−101.620, 325.573	0.304
Temporal White Matter Hyperintensities	Time × PGI × Age group old	4.069	−3.491, 11.629	0.291
	Time × PGI × Age group middle	4.637	−4.112, 13.386	0.299
Parietal White Matter Hyperintensities *	**Time × PGI × Age group old**	**53.218**	**8.311**, **98.125**	**0.020**
	Time × PGI × Age group middle	25.311	−32.633, 83.255	0.392

* Significant age group*PGI sleep* years in study interaction. Bolded: Significant parameters. Age group reference is young adults (mean aged 30 years old). ^ Results were not significant in sensitivity analyses where the same GEE model was run with a log normal transformation of total white matter hyperintensities (see Supplementary Table S2 for estimates).

**Table 4:** Contrast estimates of significant findings.

Brain Measure	Age Group Parameter Contrast	*β*	95% CI
White matter volume	Age group old	−6178.094	−32,796.006–20,438.8181
	Age group middle	−4762.517	−29,616.365–20,091.331
	Age group young	519.367	−25,783.083–36,821.690
Hippocampal volume	Age group old	−983.998	−2046.841–78.845
	Age group middle	−834.169	−1897.512–229.175
	Age group young	−749.815	1800.688–301.038
Total White Matter Hyperintensities	Age group old	597.551	−1500.780–2695.882
	Age group middle	543.379	−1715.037–2801.795
	Age group young	431.403	−1607.062–2538.867
Parietal White Matter Hyperintensities	Age group old	361.430	−184.397–907.258
	Age group middle	333.524	−239.834–906.882
	Age group young	308.213	−238.463–854.889

## Data Availability

The original contributions presented in this study are included in the article/supplementary material. Further inquiries can be directed to the corresponding author.

## Supplementary Materials

The following supporting information can be downloaded at: https://www.mdpi.com/article/10.3390/medsci14010088/s1, Table S1: ANOVA of the time × PGI interaction terms. All models were adjusted for age group, sex, education, PC1–PC4, time × Age group, and time × PC1–PC4. Table S2: Regression coefficients of the Time × PGI × Age group interactions by cognitive domains. All analyses were controlled for covariates. Table S3: Host-hoc contrast analysis of the effect of PGI on changes in cognition by age groups.
