# Supplementary material for: Polygenic Index for Sleep Duration and Brain Changes over Time
Source: Med Sci (Basel). 2026 Feb 13;14(1):88. doi: 10.3390/medsci14010088 (PMC12921902; doi:10.3390/medsci14010088)
Supplement: Supplementary file 1 [file medsci-14-00088-s001.zip › medsci-4079767-supplementary.pdf]

Supplementary Tables

Supplementary Table 1: ANOVA of the time x PGI interaction terms. All models were adjusted for age group, sex, education, PC1-PC4, time x Age group, and time x PC1-PC4

	Cognitive Domain	Parameter	Sum.Sq	Mean.Sq	NumDF	DenDF	F	p-value
No Imaging measures adjusted	Fluid Reasoning	Time x PGI	0.04431	0.04431	1	77.87	0.31	0.58
	Memory	Time x PGI	0.03104	0.03104	1	79.56	0.10	0.75
	Speed of processing	Time x PGI	0.20667	0.20667	1	79.13	2.21	0.14
	Language	Time x PGI	0.00000	0.00000	1	81.45	0.00	0.99
Total gray matter volume and mean thickness adjusted	Fluid Reasoning	Time x PGI	0.11070	0.11070	1	72.76	0.81	0.37
	Memory	Time x PGI	0.07802	0.07802	1	71.91	0.27	0.61
	Speed of processing	Time x PGI	0.12713	0.12713	1	72.21	1.80	0.18
	Language	Time x PGI	0.00509	0.00509	1	72.07	0.09	0.77

Supplementary Table 2. Regression coefficients of the Time x PGI x Age group interactions by cognitive domains. All analyses were controlled for covariates.

		Fluid Reasoning			Memory			Speed of processing			Language		
Models	parameters	Estimate	95% CI	p-value	Estimate	95% CI	p-value	Estimate	95% CI	p-value	Estimate	95% CI	p-value
No Imaging measures adjusted.	Time x PGI x Age Groups [middle]	-0.74	-16.21 – 14.74	0.93	1.08	-21.10 – 23.26	0.92	-7.84	-20.35 – 4.67	0.22	1.58	-8.17 – 11.33	0.75
	Time x PGI x Age Groups [old]	0.3	-11.61 – 12.22	0.96	5.35	-11.91 – 22.61	0.54	-7.64	-17.22 – 1.94	0.12	2.45	-5.01 – 9.91	0.52
Total gray matter volume and cortical thickness adjusted.	Time x PGI x Age Groups [middle]	-2.26	-18.00 – 13.49	0.78	0.94	-21.73 – 23.62	0.94	-9.55	-20.51 – 1.42	0.088	3.02	-7.31 – 13.35	0.56
	Time x PGI x Age Groups [old]	-1.61	-13.53 – 10.31	0.79	5.00	-12.36 – 22.37	0.57	-10.24	-18.44 – -2.03	**0.015**	2.52	-5.25 – 10.28	0.52

Supplementary Table 3. Host-hoc contrast analysis of the effect of PGI on changes in cognition by age groups.

	Age groups	Estimates	SE	df	t.ratio	p.value
No Imaging measures adjusted.	Young	5.59	4.60	80.31	1.21	0.2285
	Middle	-2.26	4.35	97.87	-0.52	0.6049
	Old	-2.05	1.70	86.38	-1.21	0.2290
Total gray matter volume and cortical thickness adjusted.	Young	**8.41**	**3.95**	**68.12**	**2.13**	**0.0370**
	Middle	-1.14	3.87	81.81	-0.29	0.7691
	Old	-1.83	1.52	75.49	-1.20	0.2337
